# Effect of continuity nursing on vision-related quality of life, anxiety, and perceived social support in patients with ocular trauma–related low vision: A propensity score–matched cohort study

**DOI:** 10.1097/MD.0000000000047318

**Published:** 2026-01-30

**Authors:** Yuxia Yin, Dan He

**Affiliations:** a Department of Ophthalmology, The Third Affiliated Hospital of Zunyi Medical University (The First People’s Hospital of Zunyi), Zunyi, China.

**Keywords:** continuity nursing, low vision rehabilitation, ocular trauma, patient-reported outcomes

## Abstract

Ocular trauma is a leading cause of acquired low vision in working-age adults and often results in long-term functional disability and psychosocial burden. Post-discharge care strategies tailored to this population remain underdeveloped. This retrospective, propensity score–matched cohort study included adult patients with ocular trauma–related low vision treated at the Third Affiliated Hospital of Zunyi Medical University (the First People’s Hospital of Zunyi) between January 2019 and December 2024. Patients who received structured continuity nursing after discharge were matched 1:1 to controls without such intervention based on 8 baseline covariates. Outcomes at 6 months included vision-related quality of life (25-item National Eye Institute Visual Function Questionnaire), anxiety symptoms (hospital anxiety and depression scale-anxiety), and perceived social support. Secondary outcomes included follow-up adherence and nursing satisfaction. Group differences were analyzed using independent *t*-tests and chi-square tests; correlation analyses explored associations among patient-reported outcomes. Among 377 screened patients with trauma-related low vision, 330 met all predefined inclusion and exclusion criteria and were eligible for further analysis. Of these, 65 patients received continuity nursing and 65 matched controls were selected for final analysis following propensity score matching. At 6-month follow-up, the intervention group reported higher 25-item National Eye Institute Visual Function Questionnaire scores (mean difference 7.6; 95% confidence interval [CI]: 4.9–10.2; *P* < .001), lower hospital anxiety and depression scale-anxiety scores (–2.6; 95% CI: –3.4 to –1.8; *P* < .001), and greater perceived social support scale scores (6.4; 95% CI: 4.1–8.7; *P* < .001). Follow-up adherence was significantly higher in the intervention group (95.4% vs 81.5%; *P* = .028), and nursing satisfaction scores were also superior (mean difference 0.8; *P* < .001). Correlation analyses revealed modest associations between visual function and social support (*r* = 0.27; *P* = .002), but no significant relationship with anxiety. Continuity nursing was associated with improved functional, psychological, and care-related outcomes in patients with ocular trauma–related low vision. These findings highlight the potential utility of structured transitional care in optimizing recovery across clinical and patient-reported domains.

## 1. Introduction

Ocular trauma remains a significant cause of acquired visual disability globally, particularly among working-age adults in low- and middle-income countries.^[1]^ In China, the burden of eye injuries has escalated in tandem with industrialization and urbanization, contributing not only to acute ophthalmic morbidity but also to long-term functional visual impairment.^[2]^ Individuals with trauma-induced low vision frequently experience persistent difficulties in daily activities, mobility, and occupational engagement, all of which compromise their health-related quality of life.^[3]^ These challenges extend well beyond visual acuity loss, often accompanied by psychological distress and reduced social participation, thereby necessitating comprehensive rehabilitative strategies beyond the acute care phase.^[4]^

Despite advances in emergency ophthalmic care and surgical techniques, structured post-discharge management for individuals recovering from ocular trauma remains largely underdeveloped.^[5]^ Continuity of care, defined as a coordinated, uninterrupted therapeutic process across care settings, serves as the overarching framework for integrated healthcare delivery. Within this framework, continuity nursing represents a nursing-led operationalization of these principles, emphasizing structured follow-up, patient education, and psychosocial support after discharge. Despite its proven benefits in chronic disease management, this form of continuity-oriented nursing remains underutilized in ophthalmic rehabilitation. Clarifying this distinction is essential, as the present study specifically evaluates continuity nursing as a practical embodiment of continuity of care tailored to post-trauma visual recovery. In real-world clinical settings, many patients are discharged without systematic rehabilitation guidance, emotional support, or follow-up mechanisms tailored to their evolving functional and psychological needs. This discontinuity in care contributes to suboptimal adherence, increased anxiety, and a diminished sense of support, ultimately undermining long-term recovery.

Moreover, current literature in ophthalmology has predominantly focused on anatomical or physiological restoration, with limited attention to patient-centered outcomes such as perceived visual functioning, psychological well-being, and social integration.^[6]^ Instruments such as the National Eye Institute Visual Function Questionnaire (NEI-VFQ-25),^[7]^ the Hospital Anxiety and Depression Scale-Anxiety (HADS-A) subscale,^[8]^ and the perceived social support scale (PSSS)^[9]^ provide validated, multidimensional frameworks to assess these outcomes, yet have rarely been employed in evaluating the broader impact of nursing interventions. The integration of such tools into post-trauma care evaluations may offer a more holistic understanding of patient recovery.

To date, few studies have investigated the longitudinal effects of continuity nursing on functional and psychosocial outcomes in patients with ocular trauma–related low vision, particularly within retrospective real-world cohorts. Furthermore, there is a paucity of research employing robust causal inference methods such as propensity score matching (PSM) to address confounding in non-randomized observational data. This study seeks to address these gaps by examining whether a structured continuity nursing intervention is associated with improved visual quality of life, reduced anxiety, and enhanced perceived social support over a 6-month period. By focusing on patient-reported outcomes in a methodologically rigorous framework, the present study aims to inform post-acute care strategies and optimize transitional rehabilitation services in ophthalmologic populations.

## 2. Methods

### 2.1. Study design and setting

This study employed a retrospective, matched cohort design to evaluate the clinical impact of continuity nursing in patients with ocular trauma–related low vision. Conducted at the Third Affiliated Hospital of Zunyi Medical University (the First People’s Hospital of Zunyi), the research analyzed data extracted from institutional electronic medical records and affiliated nursing information systems spanning from January 2019 through December 2024. All clinical protocols adhered to standardized national guidelines for ophthalmologic trauma care and postoperative follow-up, ensuring consistency across the patient population.

Patients were allocated into exposure and comparator groups based on their documented receipt of continuity nursing interventions following hospital discharge. The continuity nursing program was operationalized through the hospital’s specialist nursing department and embedded within its transitional care framework. The methodological approach and analytical framework conformed to the STROBE (Strengthening the Reporting of Observational Studies in Epidemiology) guidelines for observational research, and the overall study rationale was shaped by existing evidence on post-discharge rehabilitation care models in ophthalmology.

### 2.2. Participants and eligibility criteria

Eligible participants were retrospectively identified from the electronic medical records of the Ophthalmology Department at the Third Affiliated Hospital of Zunyi Medical University (the First People’s Hospital of Zunyi) between January 2019 and December 2024. A total of 377 adult patients with ocular trauma–related low vision were initially reviewed. Low vision was operationally defined as best-corrected visual acuity worse than log MAR 1.0 in at least 1 eye, attributable to either blunt or penetrating ocular injury. All cases were confirmed by ophthalmologists with subspecialty training, based on clinical examination and imaging data, and were treated with standard acute-phase ophthalmologic management.

Inclusion criteria were as follows: age ≥ 18 years; confirmed diagnosis of traumatic low vision; discharge from inpatient care within the defined study window; availability of complete baseline and follow-up clinical records; and completion of all 3 patient-reported outcome measures – NEI-VFQ-25, HADS-A, and PSSS – at the 6-month follow-up. Patients were excluded if they met any of the following conditions: preexisting ophthalmic comorbidities unrelated to trauma (e.g., diabetic retinopathy, glaucoma); major neurological or psychiatric disorders impairing response accuracy; cognitive or language barriers precluding completion of outcome assessments; or participation in concurrent post-discharge rehabilitation programs beyond standard care pathways.

**Figure 1.**
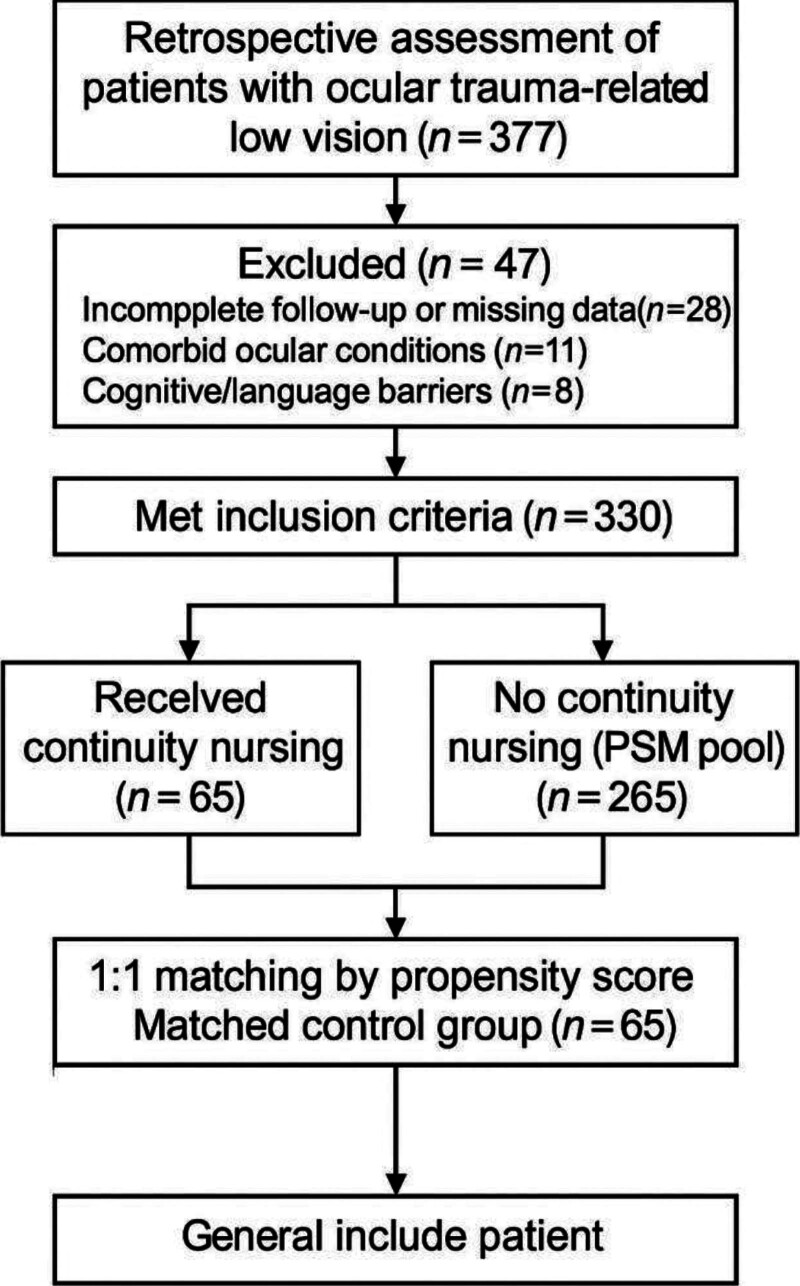
Flow diagram of included cases. PSM = propensity score matching.

After applying inclusion and exclusion criteria, 330 patients remained eligible for analysis. Following eligibility adjudication, 65 patients who had received a structured continuity nursing intervention after discharge and completed all required follow-up evaluations were assigned to the intervention group. The remaining 265 eligible patients who did not receive continuity nursing served as the potential control pool. PSM was subsequently employed to derive a balanced control group for comparative analysis, as detailed below. The full patient selection process is summarized in Figure 1.

### 2.3. Intervention: continuity nursing protocol

The intervention under investigation was a structured continuity nursing program specifically designed for patients recovering from ocular trauma. This protocol was initiated upon discharge and implemented by certified ophthalmic nurse specialists within a transitional care model coordinated by the hospital’s nursing department. The intervention encompassed multiple components, including individualized discharge planning, scheduled post-discharge telephone consultations (within 72 hours), bi-weekly remote monitoring (via telephone), and structured follow-up visits at 1, 3, and 6 months. Educational modules addressing visual rehabilitation, injury prevention, medication adherence, and psychosocial coping were delivered in modular format and tailored to patient-specific needs and literacy levels.

Additionally, patients assigned to the continuity nursing pathway received access to a dedicated communication channel staffed by senior nursing personnel, allowing for real-time problem resolution and emotional support. A case management approach ensured consistency, with the same nurse practitioner overseeing each patient’s longitudinal care. All interventions were documented in the institutional nursing follow-up registry. The fidelity of the program was monitored through quarterly quality audits and internal peer review. Control group patients received routine discharge instructions and standard outpatient care as determined by attending physicians, with no formalized nursing continuity structure beyond hospital discharge. The intensity and scope of the continuity nursing intervention were standardized across cases and detailed in institutional nursing protocols to minimize provider-dependent variability.

### 2.4. Outcome measures

The primary outcome measures comprised 3 validated patient-reported instruments capturing distinct but interrelated domains of recovery: vision-related quality of life, psychological distress, and perceived social support. Visual function was assessed using the Chinese-translated version of the NEI-VFQ-25, a well-established tool for quantifying functional vision impairment across domains such as near and distance activities, dependency, and social functioning. Anxiety symptoms were evaluated via the HADS-A, a 7-item instrument widely adopted in ophthalmologic and rehabilitation research due to its brevity and psychometric robustness. Social support perception was quantified using the PSSS, which evaluates support from family, friends, and significant others on a 5-point Likert-type scale. All instruments were administered in person or remotely at baseline (prior to discharge) and again at 6 months post-discharge, by trained assessors blinded to group allocation.

Secondary outcomes focused on care process indicators and patient experience metrics. Adherence to scheduled follow-up was defined dichotomously as completion of the 6-month reassessment and recorded from institutional follow-up logs. Patient satisfaction with nursing care was collected using a 5-point Likert scale adapted from national service evaluation benchmarks, with higher scores indicating greater satisfaction across domains such as accessibility, emotional support, and informational clarity. All outcome measures were pre-specified in the study protocol and uniformly applied to both intervention and control groups. Continuous outcomes were expressed as mean ± standard deviation, while categorical data were reported as absolute frequencies and percentages.

### 2.5. Ethics approval

This study was reviewed and approved by the Institutional Ethics Committee of the Third Affiliated Hospital of Zunyi Medical University (the First People’s Hospital of Zunyi; Approval No. 2019-1-43). Given the retrospective nature of the study and the use of routinely collected de-identified clinical data, the requirement for individual informed consent was formally waived by the committee. All procedures were conducted in accordance with the ethical standards outlined in the Declaration of Helsinki and its subsequent revisions, and followed the ethical principles endorsed by the Chinese Medical Association for human participant research.

Data extraction, patient identification, and outcome assessment were performed within the secure hospital data environment, and no data were accessed outside the institutional firewall. Personal identifiers were removed at the point of analysis, and access to raw data was restricted to study personnel who had completed institutional research ethics training. To ensure full transparency and adherence to international reporting norms, the study followed the Strengthening the Reporting of Observational Studies in Epidemiology (STROBE) guidelines for cohort studies. No patient or public involvement occurred in the design, execution, or interpretation of this analysis.

### 2.6. Statistical analysis

To minimize baseline confounding in this observational cohort, PSM was applied using a logistic regression model that incorporated 8 prespecified covariates: age, sex, education level, trauma type (penetrating vs blunt), laterality, baseline visual acuity (log MAR), NEI-VFQ-25 score, and urban residency status. A nearest-neighbor matching algorithm with a caliper of 0.2 standard deviations of the logit of the propensity score was employed, without replacement, in a 1:1 ratio. Post-matching balance was assessed using standardized mean differences (SMDs), with an SMD < 0.1 indicating acceptable covariate balance. Variables such as occupational status, socioeconomic level, and trauma severity were not included due to inconsistent documentation across medical records. To evaluate the robustness of the matching procedure, sensitivity checks were performed using alternative caliper widths (0.1 and 0.3 of the standard deviation of the logit of the propensity score) and yielded comparable results with SMDs remaining <0.1 across all covariates. These analyses support the stability of covariate balance and the reliability of the matched sample for outcome comparison.

Continuous variables were summarized using means and standard deviations and compared using independent-sample *t*-tests, as the matched pairs represented distinct individuals rather than repeated measures. Categorical variables were compared using chi-square or Fisher’s exact tests as appropriate. Categorical variables were compared using chi-square tests or Fisher’s exact tests when expected cell counts were <5. Effect sizes were reported as mean differences with 95% confidence intervals (CIs), and Cohen’s *d* was calculated for between-group comparisons to estimate standardized effect magnitude. Subgroup analyses for the primary outcome (NEI-VFQ-25 score) were performed across age, education level, and trauma type strata using stratified independent *t*-tests. These analyses were exploratory in nature, conducted without adjustment for multiple comparisons or inclusion of formal interaction terms, and were intended to describe potential variability in intervention effects rather than infer causal subgroup differences. Pearson correlation coefficients were calculated to examine the associations among NEI-VFQ-25, HADS-A, and PSSS scores at 6-month follow-up. All tests were 2-tailed, and a *P*-value < .05 was considered statistically significant. Although no a priori sample size estimation was conducted due to the retrospective design, a post hoc power analysis was performed based on the observed between-group differences in the 3 primary outcomes (NEI-VFQ-25, HADS-A, and PSSS). The corresponding effect sizes (Cohen’s *d*) were 0.97, 0.90, and 0.91, yielding statistical power estimates of 0.87, 0.85, and 0.86, respectively (α = 0.05, 2-tailed; n = 65 per group). These results indicate that the matched cohort provided sufficient power to detect clinically meaningful between-group differences across all primary endpoints.

## 3. Results

### 3.1. Patient selection and baseline covariate balance after matching

A total of 377 patients with ocular trauma–related low vision were retrospectively screened. Following application of predefined inclusion and exclusion criteria, 330 patients were deemed eligible. Of these, 65 patients who received structured continuity nursing and completed all follow-up assessments formed the intervention group. The remaining 265 eligible patients who did not receive continuity nursing were evaluated as the control pool. Propensity scores were estimated using logistic regression based on 8 baseline covariates: age, sex, education level, trauma type (penetrating vs blunt), laterality, baseline visual acuity (log MAR), NEI-VFQ-25 score, and urban residency. A 1:1 nearest-neighbor matching algorithm without replacement and with a caliper of 0.2 standard deviations was applied. Sixty-five matched controls were successfully identified, yielding a final analytic cohort of 130 patients (Table 1, Fig. 1).

**Table 1:** Baseline characteristics of patients before and after propensity score matching.

Variable	Before matching			After matching		
	Intervention	Control	*P*-value	Intervention	Control	SMD
Age (yr), mean (SD)	47.8 (13.1)	45.9 (12.7)	.23	47.8 (13.1)	47.5 (12.9)	0.02
Male sex, n (%)	52 (80.0%)	255 (81.7%)	.75	52 (80.0%)	53 (81.5%)	0.04
Education level, n (%)						
Middle school or below	27 (41.5%)	116 (37.2%)	.36	27 (41.5%)	28 (43.1%)	0.03
High school	24 (36.9%)	117 (37.5%)		24 (36.9%)	23 (35.4%)	
College or above	14 (21.5%)	79 (25.3%)		14 (21.5%)	14 (21.5%)	
Injury type, n (%)						
Penetrating	39 (60.0%)	164 (52.6%)	.19	39 (60.0%)	40 (61.5%)	0.03
Blunt trauma	26 (40.0%)	148 (47.4%)		26 (40.0%)	25 (38.5%)	
Laterality, n (%)						
Unilateral	50 (76.9%)	247 (79.2%)	.66	50 (76.9%)	51 (78.5%)	0.04
Bilateral	15 (23.1%)	65 (20.8%)		15 (23.1%)	14 (21.5%)	
Visual acuity (log MAR), mean (SD)	1.23 (0.34)	1.15 (0.38)	.04	1.23 (0.34)	1.22 (0.35)	0.03
NEI-VFQ-25 score (baseline), mean (SD)	57.2 (8.1)	60.3 (9.0)	.02	57.2 (8.1)	57.5 (8.2)	0.04
HADS-A score (baseline), mean (SD)	9.1 (2.7)	8.1 (3.0)	.03	9.1 (2.7)	9.0 (2.6)	0.02
PSSS score (baseline), mean (SD)	54.3 (7.0)	56.5 (6.9)	.06	54.3 (7.0)	54.1 (6.8)	0.02
Urban residence, n (%)	44 (67.7%)	234 (75.0%)	.19	44 (67.7%)	45 (69.2%)	0.03

Propensity score matching was performed using 1:1 nearest neighbour matching without replacement and a caliper width of 0.2 standard deviations of the logit of the propensity score, based on baseline covariates including age, sex, education, injury type, visual acuity, laterality, NEI-VFQ-25, and residency.

Continuous variables are presented as mean (standard deviation); categorical variables are presented as number (%).

HADS-A = hospital anxiety and depression scale–anxiety, log MAR = logarithm of the minimum angle of resolution, NEI-VFQ-25 = National Eye Institute Visual Function Questionnaire-25, PSSS = perceived social support scale, SD = standard deviation, SMD = standardized mean difference.

**Figure 2.**
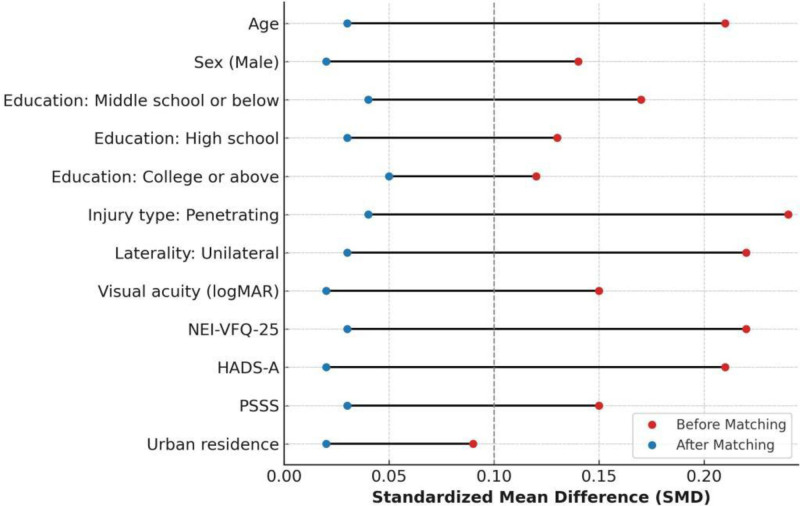
Comparison of standardized mean differences before and after matching across variables. HADS-A = hospital anxiety and depression scale–anxiety, NEI-VFQ-25 = National Eye Institute Visual Function Questionnaire-25, PSSS = perceived social support scale, SMD = standardized mean differences.

Prior to matching, statistically significant imbalances were observed in baseline visual acuity (mean log MAR 1.23 vs 1.15; *P* = .04), NEI-VFQ-25 score (57.2 ± 8.1 vs 60.3 ± 9.0; *P* = .02), and HADS-A score (9.1 ± 2.7 vs 8.1 ± 3.0; *P* = .03), with modest differences also noted in urban residency and injury type. Following matching, all covariates were well balanced between groups, with SMDs below the accepted threshold of 0.1 (Fig. 2). Mean age was 47.8 years in the intervention group and 47.5 years in controls; male patients accounted for 80.0% and 81.5%, respectively. Education level, trauma laterality, and baseline scores for NEI-VFQ-25, HADS-A, and PSSS were closely matched (Table 1). These results confirmed the adequacy of the matching procedure and established baseline comparability between groups prior to outcome evaluation.

### 3.2. Group differences in visual function, anxiety, and social support at follow-up

**Figure 3.**
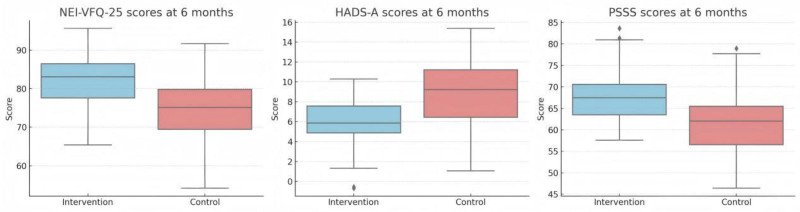
Comparison of NEI-VFQ-25, HADS-A, and PSSS scores between Intervention and Control groups at 6 months. HADS-A = hospital anxiety and depression scale–anxiety, NEI-VFQ-25 = National Eye Institute Visual Function Questionnaire-25, PSSS = perceived social support scale.

At the 6-month follow-up, all 3 outcome measures – NEI-VFQ-25, HADS-A, and PSSS – showed statistically and clinically significant differences between the continuity nursing group and the matched controls (Fig. 3). Independent *t*-tests were conducted for each outcome variable. The intervention group demonstrated a higher mean NEI-VFQ-25 score (81.7 ± 7.4) compared to the control group (74.1 ± 8.2), with a mean difference of 7.6 points (95% CI: 4.9–10.2; *P* < .001; Cohen’s *d* = 0.96). This indicates a substantial improvement in vision-related quality of life associated with continuity nursing.

In terms of psychological outcomes, the intervention group had significantly lower HADS-A scores at follow-up (5.7 ± 2.2) than the control group (8.3 ± 2.9), with a mean difference of–2.6 (95% CI: –3.4 to –1.8; *P* < .001; Cohen’s *d* = 1.05), indicating reduced anxiety levels. Similarly, perceived social support was higher in the intervention group (PSSS: 67.9 ± 6.1) than in controls (61.5 ± 7.0), with a mean difference of 6.4 (95% CI: 4.1–8.7; *P* < .001; Cohen’s *d* = 0.98). Collectively, these results suggest a consistent and favorable impact of continuity nursing on both functional and psychosocial outcomes in patients with post-traumatic low vision.

### 3.3. Secondary outcomes: follow-up adherence and patient satisfaction

**Figure 4.**
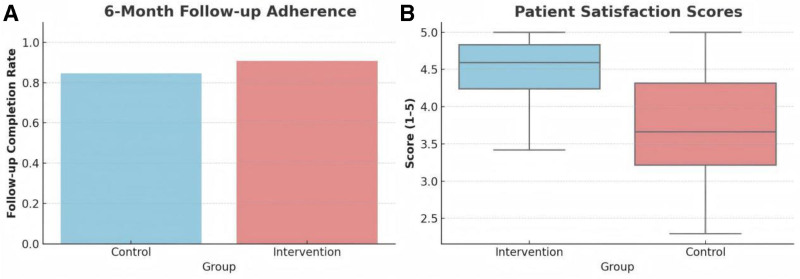
Comparative analysis of (A) 6-month follow-up adherence and (B) patient satisfaction scores between intervention and control groups.

In addition to improvements in clinical outcomes, continuity nursing also demonstrated advantages in secondary care-related measures (Fig. 4). At the 6-month follow-up, adherence to scheduled assessment was significantly higher in the intervention group (62/65; 95.4%) compared to the control group (53/65; 81.5%; *P* = .028, χ² test). This suggests that patients receiving structured follow-up and transitional support were more likely to engage with post-discharge care.

Satisfaction with nursing services was also evaluated using a 5-point Likert scale, with higher scores indicating better subjective experience. The intervention group reported significantly greater satisfaction (mean score 4.4 ± 0.5) than the control group (3.6 ± 0.7), with a mean difference of 0.8 points (95% CI: 0.5–1.1; *P* < .001; Cohen’s *d* = 1.29). These findings reinforce the added value of continuity nursing in enhancing not only functional recovery, but also patient experience and healthcare engagement (Fig. 4).

### 3.4. Subgroup analysis of intervention effects across demographic and clinical characteristics

**Figure 5.**
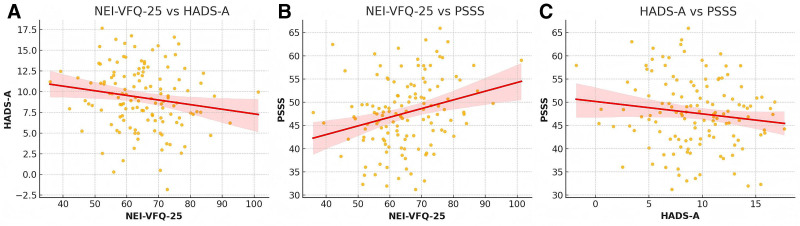
Scatter plot an analysis of the relationships among NEI-VFQ-25, HADS-A, and PSSS scores. (A) NEI-VFQ-25 versus HADS-A. (B) NEI-VFQ-25 versus PSSS. (C) HADS-A versus PSSS. HADS-A = hospital anxiety and depression scale–anxiety, NEI-VFQ-25 = National Eye Institute Visual Function Questionnaire-25, PSSS = perceived social support scale.

Exploratory subgroup analyses were performed to examine within-stratum differences in NEI-VFQ-25 outcomes between intervention and control groups across predefined demographic and clinical categories (Table 2, Fig. 5). Across all 6 subgroups, patients receiving continuity nursing consistently demonstrated higher NEI-VFQ-25 scores at 6 months than their matched counterparts.

**Table 2:** Subgroup stratified comparison.

Subgroup	Mean difference	95% CI	*P*-value
Age ≥ 60	9.2	6.0–12.3	.002
Age < 60	6.3	3.2–9.4	.015
Education ≤ middle school	8.4	5.1–11.7	.004
Education ≥ high school	5.9	2.7–9.1	.026
Penetrating trauma	9.0	5.8–12.2	.001
Blunt trauma	6.2	2.9–9.5	.019

CI = confidence interval.

Within the age subgroups, the mean between-group difference was 9.2 points (95% CI: 6.0–12.3; *P* = .002) among patients aged ≥60 years and 6.3 points (95% CI: 3.2–9.4; *P* = .015) among those younger than 60 years. Similar within-stratum patterns were observed across educational levels and trauma types: among patients with lower education (≤middle school), the mean difference was 8.4 points (95% CI: 5.1–11.7), compared with 5.9 points (95% CI: 2.7–9.1) in those with higher education. Among patients with penetrating trauma, the mean difference was 9.0 points (95% CI: 5.8–12.2), whereas in blunt trauma cases it was 6.2 points (95% CI: 2.9–9.5).

These subgroup findings reflect consistent within-stratum improvements associated with continuity nursing. No formal statistical tests for interaction or between-subgroup differences were performed, and the results should be interpreted as descriptive trends rather than confirmatory evidence of effect modification.

### 3.5. Correlation between visual function, anxiety, and perceived social support

To explore potential interrelationships among patient-reported outcomes, Pearson correlation analyses were performed between NEI-VFQ-25, HADS-A, and PSSS scores at the 6-month follow-up (Fig. 5). A modest positive correlation was observed between NEI-VFQ-25 and PSSS (*r* = 0.27, *P* = .002), suggesting that patients with better visual function reported stronger perceived social support. Conversely, NEI-VFQ-25 scores showed a weak negative correlation with anxiety scores (HADS-A: *r* = −0.16, *P* = .071), which did not reach statistical significance.

In addition, PSSS and HADS-A scores were negatively correlated (*r* = −0.13, *P* = .128), although again not statistically significant. These findings may indicate a mutually reinforcing pattern whereby patients with stronger social connections and reduced psychological distress are more likely to engage in visual rehabilitation, or conversely, that improved functional vision itself contributes to emotional well-being and interpersonal confidence. As this analysis is correlational and exploratory in nature, causal inferences cannot be drawn (Fig. 5).

## 4. Discussion

This propensity score–matched cohort study demonstrated that continuity nursing was associated with significantly improved vision-related quality of life, reduced anxiety symptoms, and enhanced perceived social support among patients with ocular trauma–related low vision over a 6-month follow-up. These findings, derived from rigorously matched real-world cohorts and assessed through validated patient-reported outcome measures, suggest that a structured post-discharge care model may yield meaningful benefits in both functional recovery and psychosocial well-being for this vulnerable population. The consistency and magnitude of group differences, particularly in NEI-VFQ-25 and HADS-A scores, point to a potentially multifaceted therapeutic effect extending beyond routine clinical rehabilitation.

The observed between-group differences in NEI-VFQ-25 scores – exceeding the minimally important difference thresholds reported in previous ophthalmic studies – indicate clinically relevant improvement in vision-related daily functioning. While much of the literature on ocular trauma has focused on anatomical outcomes or surgical endpoints,^[10]^ few studies have systematically explored the long-term subjective experience of visual recovery.^[11]^ Moreover, anxiety reduction and increased perceived social support among patients receiving continuity nursing suggest that the intervention may act through pathways involving emotional regulation and interpersonal reinforcement. This aligns with previous evidence from chronic illness populations, such as stroke and diabetes, where transitional care models have demonstrated efficacy in alleviating psychological distress and enhancing adherence.^[12]^ In the ophthalmic context, however, continuity nursing remains understudied, and the present findings may thus offer an important contribution to an emerging care paradigm.^[13,14]^

The potential mechanisms through which continuity nursing exerts its effects are likely multifactorial.^[15]^ Regular follow-up contacts, reinforcement of self-management behaviors, and empathetic communication by designated care staff may collectively foster a sense of security and accountability, which in turn enhances psychological resilience and care engagement. The observed positive correlation between visual function and social support, though modest in strength, may reflect the reciprocal relationship between emotional resources and functional independence. Conversely, the weak and statistically nonsignificant inverse correlation between anxiety and visual function suggests that while distress may accompany functional limitation, its amelioration may depend on factors beyond visual status alone. It remains plausible that continuity nursing improves anxiety levels through mechanisms independent of vision per se, such as relational continuity or perceived responsiveness of the care system.

Several methodological strengths underpin the robustness of our findings. PSM was employed to mitigate selection bias and baseline imbalances inherent to retrospective observational designs. Standardized differences post-matching were consistently below conventional thresholds, and visual inspection confirmed satisfactory overlap in propensity distributions. The use of validated instruments spanning functional, psychological, and social domains strengthens construct validity and permits multidimensional assessment of intervention impact. Additionally, our inclusion of secondary outcomes such as follow-up adherence and patient satisfaction provides further granularity on the acceptability and feasibility of continuity nursing in real-world clinical practice.

Nonetheless, this study is not without limitations. The retrospective design precludes definitive causal inference, and despite matching, residual confounding by unmeasured variables, such as health literacy or motivation, cannot be excluded. Selection bias may also be present, as patients willing to participate in structured care programs may differ systematically from those who decline. Furthermore, outcome assessments relied on self-reported measures, which are inherently subject to response and recall biases. The 6-month follow-up window, while sufficient to detect short- to mid-term effects, does not capture the sustainability or evolution of intervention benefits over time. Additionally, socioeconomic status, occupational background, and trauma severity were not consistently documented and thus could not be incorporated into the propensity score model. Sensitivity checks with alternative caliper widths (0.1–0.3 SD) yielded similar covariate balance, suggesting robustness of the main findings despite these data limitations. Future studies using prospective or randomized designs, longer follow-up, and implementation metrics would be valuable to validate and extend these findings. Finally, the subgroup analyses were exploratory and performed without multiplicity correction or interaction testing. Therefore, the apparent variations in treatment effects across age, education, and trauma type strata should be interpreted cautiously as hypothesis-generating rather than confirmatory findings.

In conclusion, our findings suggest that continuity nursing may serve as a beneficial adjunct to standard post-trauma care for patients with ocular trauma–related low vision, with positive implications for both vision-related function and psychosocial health. By addressing unmet needs in the post-discharge phase, such interventions may help bridge existing gaps in ophthalmic care delivery. Further research is warranted to refine these models and explore their generalizability across different care settings and patient subgroups.

## 5. Conclusion

In this propensity score–matched cohort study of patients with ocular trauma–related low vision, continuity nursing was associated with significantly greater improvements in vision-related quality of life, anxiety reduction, and perceived social support over a 6-month follow-up period. These findings underscore the potential value of structured post-discharge care models in addressing functional and psychosocial needs beyond the acute phase of ocular injury. While causality cannot be definitively established, the consistency of effects across multiple patient-reported outcomes and care process indicators supports the integration of continuity-focused strategies into routine ophthalmic rehabilitation. Future prospective research is warranted to validate these observations, explore underlying mechanisms, and determine the long-term sustainability of benefit across diverse clinical settings.

## Author contributions

**Conceptualization:** Yuxia Yin, Dan He.

**Formal analysis:** Yuxia Yin.

**Investigation:** Yuxia Yin.

**Methodology:** Dan He.

**Project administration:** Dan He.

**Writing – original draft:** Yuxia Yin.

**Writing – review & editing:** Dan He.
